# Atypical Case of Three Dental Implants Displaced into the Maxillary Sinus

**DOI:** 10.1155/2015/896423

**Published:** 2015-10-08

**Authors:** João Felipe Bonatto Bruniera, Yara Teresinha Corrêa Silva-Sousa, Paulo Esteves Pinto Faria

**Affiliations:** University of Ribeirão Preto (UNAERP), Avenue Costábile Romano 2201, 14096-900 Ribeirão Preto, SP, Brazil

## Abstract

Oral rehabilitation with dental implants has become a routine treatment in contemporary dentistry. The displacement of dental implants into the sinus membrane, a complication related to the maxillary sinus, is one of the most common accidents reported in the literature. The treatment for this complication is the surgical removal of the implant. A 60-year-old woman with three dental implants displaced into the maxillary sinus (one implant displaced into the left maxillary sinus and two implants displaced into the right maxillary sinus) underwent surgery for removal of the implants. The surgery to remove the implants was performed under local anesthesia through the Caldwell-Luc technique. The patient was subsequently administered antibiotic, anti-inflammatory, and analgesic drugs. The patient returned 7 days after the surgery for suture removal and is being regularly monitored to determine whether future rehabilitation of the edentulous area is necessary. In conclusion, surgical removal of the dental implant displaced into the maxillary sinus is the treatment of choice. This technique is appropriate because it allows the use of local anesthesia and provides direct visualization for the removal of the implants.

## 1. Introduction

Oral rehabilitation with dental implants has become a routine treatment in contemporary dentistry and is generally considered a safe surgical procedure with a high success rate. However, as with other surgical procedures, this type of surgery can lead to complications, which should be taken into consideration. In some cases, complications are resolved without major problems; in contrast, there are more serious complications that can compromise a patient's health [1].

The implant complications related to surgery include hemorrhage, neurosensory disorders caused by nerve injury, damage to adjacent teeth, mucoperiosteal flap dehiscence, and exposure of bone and implant. Complications involving the maxillary sinus include perforation of the sinus membrane, bleeding from the sinus cavity, displacement of implants or other materials into the maxillary sinus, and postoperative sinusitis [2]. One of the most likely areas for transsurgical complications is the posterior maxilla. This region is characterized by soft bone with no cortical portions and a large trabecular portion, which will probably destabilize the implant and could cause it to fail. Typically, the resorption of the alveolar ridge, sinus pneumatization, and inflammatory reaction in the peri-implant tissues may promote the displacement of the implant into the sinus cavity [3].

New techniques to address these complications have emerged with the evolution of dentistry, such as the elevation of the maxillary sinus floor and use of short implants. However, this type of accident is still rather found and often cited in the literature. To avoid these complications, presurgical planning is mandatory to evaluate the presence of a vertical bone deficiency that may contraindicate conventional implant placement, particularly implant migration inside the sinus cavity. The most common maxillary sinus complication found in the literature is the displacement of implants for which the lead treatment procedure is the surgical removal of the implant [3]. Nevertheless, various treatment modalities have been employed to deal with this complication from leaving the migrated implant untreated under monitoring to endoscopic transnasal procedures or a conventional Caldwell-Luc technique. Different theories have been proposed to explain the mechanism by which implant migration occurs [4]. The most important complication to consider is the inadequate treatment in the posterior maxilla region. This fact could be influenced significantly in implant displacement for the maxillary sinus [5].

The aim of this study was to report the case of removing implants displaced into the maxillary sinus.

## 2. Case Report

**Figure 1:**
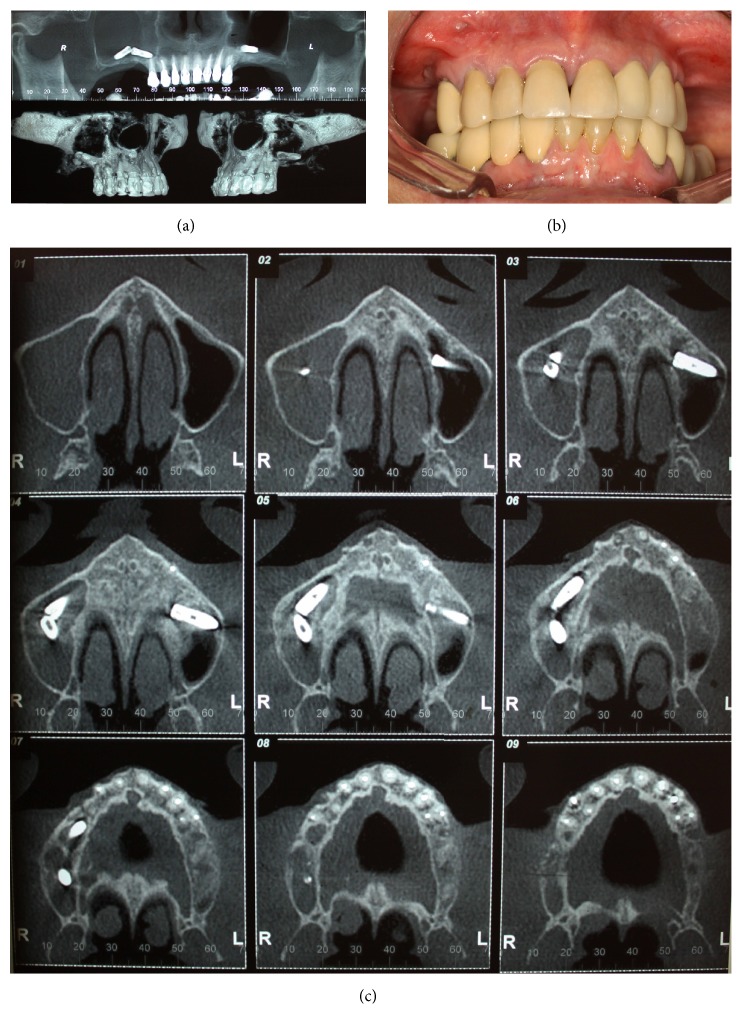
(a) A panoramic and a tridimensional reconstruction CT scan showing the implants displaced into the maxillary sinus cavity with a significant opacification of the right maxillary sinus; however, the left sinus does not present the opacification. (b) Photography showing the frontal clinical view. (c) A CT scan in axial sections showing implant displacement into the maxillary sinus cavity with a significant opacification of the right maxillary sinus. The left sinus does not present the opacification.

A 60-year-old woman was referred to the Department of Oral and Maxillofacial Surgery of the University of Ribeirão Preto (UNAERP) because she had displacement of three dental implants into the maxillary sinus, two of which were in the right sinus and one was in the left sinus. According to the report, the patient underwent surgery for implant placement in the maxilla 8 months ago. Three months after the surgery, she began to report pain and felt pressure in the premolar region, particularly on the right side. The patient also had pain on palpation of the anterior wall of the right maxillary sinus, with no sign of oroantral fistula (Figure 1(b)).

After examination of panoramic radiography, two similar images of dental implants were noted in the right maxillary sinus with evidence of sinusitis, including opacification of the maxillary sinus. On the left sinus, the same image pattern, corresponding to a dental implant, was found; however, no sign of sinusitis was found. Computed tomography (CT) showed implants displaced into the maxillary sinus cavity with a significant opacification of the right maxillary sinus; however, the left sinus did not present the opacification; only the implant did (Figures 1(a) and 1(c)).

**Figure 2:**
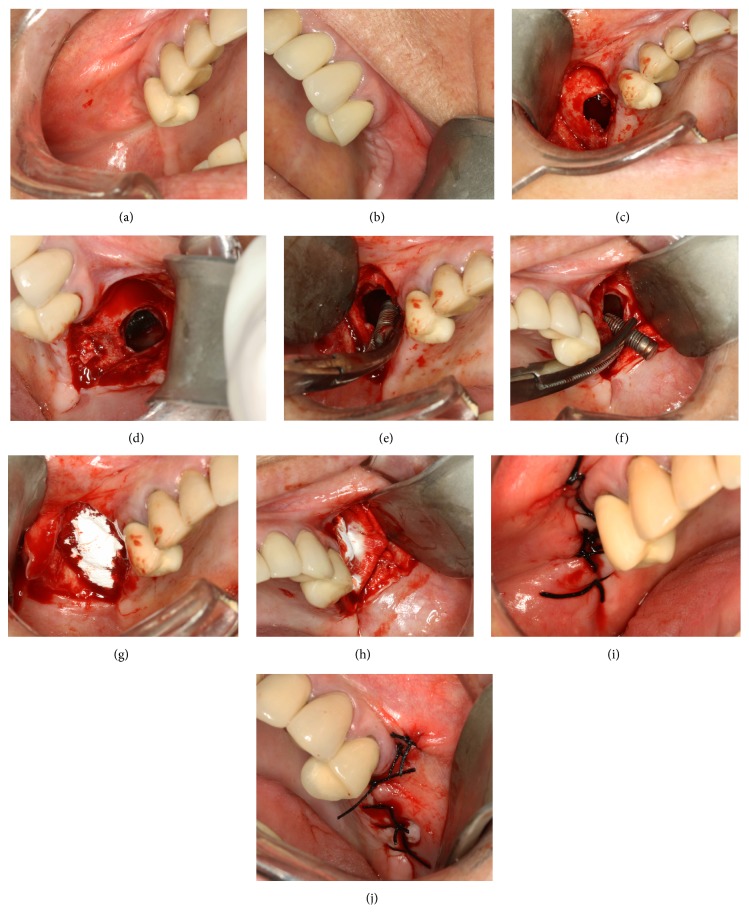
(a) Clinical photograph showing the clinical view of the right side. (b) Clinical photograph showing the clinical view of the left side. (c) Clinical photograph showing membrane perforation through the right bone window to gain full access into the sinus cavity. (d) Clinical photograph showing membrane perforation through the left bone window to gain full access into the sinus cavity. (e) Clinical photograph showing the implant removed with a Kelly clamp in the right sinus. (f) Clinical photograph showing the implant removed with a Kelly clamp in the left sinus. (g) Clinical photograph showing a porcine collagen membrane (Geistlich Bio-Gide, Switzerland) installed in the lateral bone window to protect the right sinus cavity. (h) Clinical photograph showing a porcine collagen membrane (Geistlich Bio-Gide, Switzerland) installed in the lateral bone window to protect the left sinus cavity. (i) Clinical photograph showing the surgical flap sutured (right side). (j) Clinical photograph showing the surgical flap sutured (left side).

**Figure 3:**
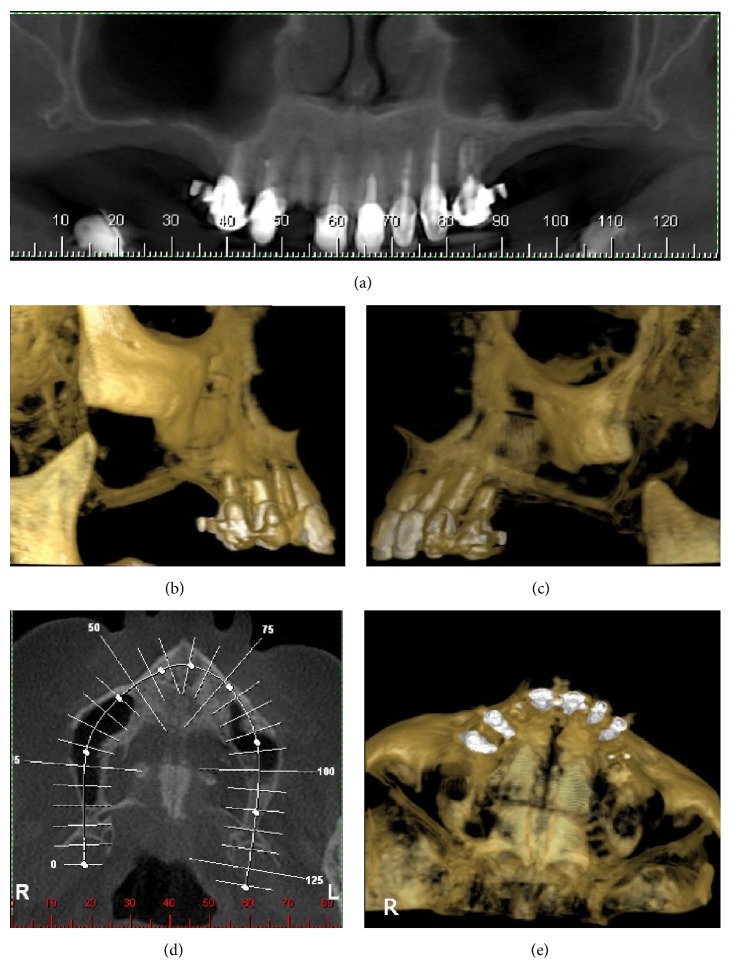
(a) Panoramic view showing a healthy maxillary sinus cavity after 12 months. (b), (c) Tridimensional reconstruction computed tomography scan showing preserved bony structures. (d) and (e) Occlusal slice showing unobstructed maxillary sinus cavities.

*Surgical Procedure*. The patient was operated under local anesthesia (Figures 2(a) and 2(b)). An oral antibiotic prophylaxis (amoxicillin + clavulanate, 2.0 g) was administered 1 h prior to start of the procedure. The surgical intervention began with the Caldwell-Luc access bilaterally and the elevation of a trapezoidal full-thickness mucoperiosteal flap. The buccal aspect of the flap was raised to access the maxillary sinus bony wall. A low-speed straight hand piece with a circular diamond bur was used to perform spherical osteotomy. The Schneiderian membrane was perforated and removed through the bone window to gain full access into the sinus cavity (Figures 2(c) and 2(d)). Immediately thereafter, the implants were identified and removed with a surgical aspirator and a Kelly clamp (Figures 2(e) and 2(f)). In the right maxillary sinus, the inflammatory tissue was removed and the surgical field was irrigated with sterile saline in both maxillary sinuses. After irrigation, a porcine collagen membrane (Geistlich Bio-Gide, Switzerland) was installed in the lateral bone window to protect the sinus cavity (Figures 2(g) and 2(h)). After irrigation of the surgical field with sterile saline, the surgical flap was sutured (Figures 2(i) and 2(j)), and an antibiotic therapy with amoxicillin and clavulanate (1.0 g) was prescribed in association with a nonsteroidal anti-inflammatory drug every 8 h for 7 days. Chlorhexidine mouthwashes were used along with usual oral hygiene for 7 days. The patient was instructed to avoid using a mucosupported prosthesis for 15 days to prevent suture dehiscence. The postoperative recovery was uneventful. After 7 days, the patient returned to the university for suture removal and clinical examination. Twelve months after the procedure, a CT scan showed the maxillary sinus without the opacification, with no sign of sinusitis (Figures 3(a)–3(e)). The patient is being regularly monitored for future rehabilitation of the edentulous area. The Helsinki declaration was followed in this case report.

## 3. Discussion

The displacement of dental implants, like that of any other foreign body into the interior of the maxillary sinuses, can cause reactions of infection in the maxillary sinuses, which may extend to other cavities, such as the paranasal, orbital, and intracranial cavities, thus, aggravating the patient's condition [6].

Thus, the treatment always preferred is the surgical removal of these foreign bodies. The surgical technique used in this study was to remove the implants using the Caldwell-Luc approach, which requires the removal of a bone window of the lateral wall of the maxillary sinus for a direct approach to this cavity [7]. Other techniques are also reported in the literature, such as the removal of foreign bodies from the maxillary sinus through the nasal cavity [8], removal by endoscopic probe through the side wall of the maxillary sinus [9], and creation of a bone lid of the anterior wall of the maxillary sinus [10].

A classification by Felisati et al., 2013 [11] was conducted to simplify and introduce a surgical protocol. In this paper, the authors classified the sinonasal complications resulting from dental treatment and suggested functional endoscopic sinus surgery (FESS), implant removal, or oroantral communication (OAC) repair for treatment of the implant dislocation with sinusitis. In the present paper, surgery was performed using the Caldwell-Luc approach to remove the implants and the inflammatory tissue because this is a simple approach, which can be reproduced by any clinician with surgical experience. The FESS procedure was not used because of hospitalization and lack of OAC in the maxillary sinus. After 12 months, the CT scan showed a maxillary sinus without opacification, suggesting a normal patency of the maxillary sinus.

Techniques involving paranasal probes through the sinuses to preserve the pairs of maxillary sinuses are more complex to use because these techniques usually require use of general anesthesia. In addition, when the foreign body is located in the anterior and inferior regions of the maxillary sinus, it is difficult to remove the foreign body with probes [10]. Therefore, in this case, we adopted a technique of taking a direct approach from the sinuses, which provides better visualization of the area addressed during the removal of the implants.

## 4. Conclusion

It can be concluded that the Caldwell-Luc surgical approach is a simple approach for the removal of inwardly displaced maxillary sinus implants. The technique adopted was appropriate because of the possibility of performing the surgical approach under local anesthesia and direct visualization of all procedures during removal of the implants. To avoid systemic problems, the implants should be removed as soon as possible or the patient should be referred to a maxillofacial surgeon to remove the implant. In cases associated with sinusitis, an appointment with an otorhinolaryngologist should be made to assess the health of all paranasal sinuses.
